# Supplementary material for: Morphological Medial Gastrocnemius Muscle Growth in Ambulant Children with Spastic Cerebral Palsy: A Prospective Longitudinal Study
Source: J Clin Med. 2023 Feb 16;12(4):1564. doi: 10.3390/jcm12041564 (PMC9963346; doi:10.3390/jcm12041564)
Supplement: Supplementary file 1 [file jcm-12-01564-s001.zip › jcm-2220634-supplementary.pdf]

Supplementary Materials

Figure S1: Observed individual profiles for the (A) absolute and (B) normalized muscle morphology for (i) the total group of children with SCP (blue=GMFCS level I, dark red=level II and pink=level III), (ii) children with GMFCS level I, (iii) children with GMFCS level II and (iv) children with GMFCS level III. *SCP, spastic cerebral palsy; GMFCS, gross motor function classification system; MV, muscle volume; ml, milliliter; CSA, anatomical cross-sectional area; mm, millimeter; ML, muscle belly length; n, normalized; kg, kilogram; m, meters.*

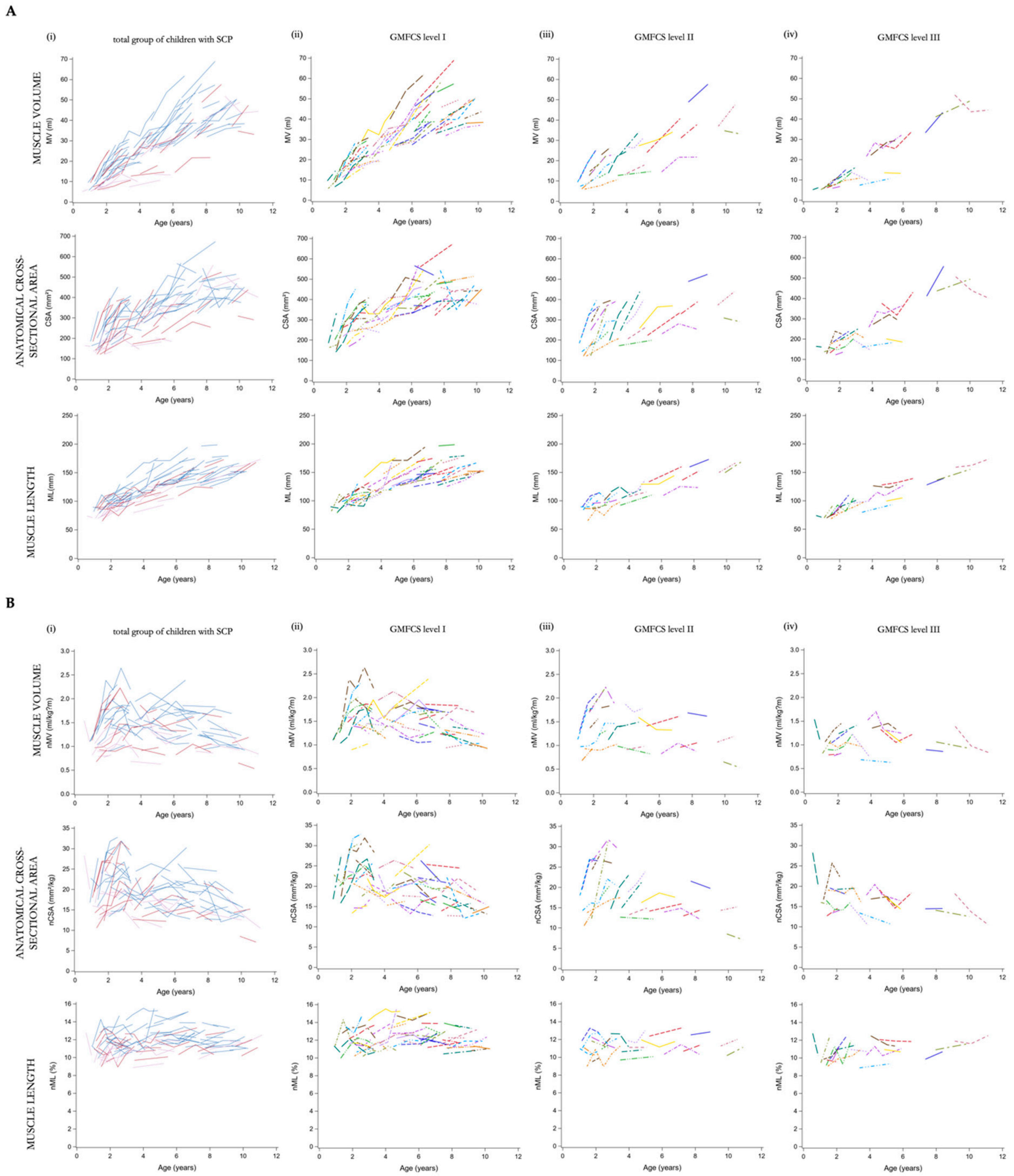

Figure S2

A: Reference dataset for absolute morphological muscle outcomes and **B:** Reference dataset for normalized morphological muscle outcomes (i) in addition to the predicted trajectories for the children with GMFCS levels I (ii) (blue) and GMFCS level II-III (iii) (red). For the TD children, the individual observed values (grey dots) and median with IQR per one year–age group (green boxplots) are presented. *TD, typically developing children; SCP, spastic cerebral palsy; GMFCS, gross motor function classification system; IQR, interquartile range; MV, muscle volume; ml, milliliter; CSA, anatomical cross-sectional area; mm, millimeter; ML, muscle belly length; n, normalized; kg, kilogram; m, meters.*

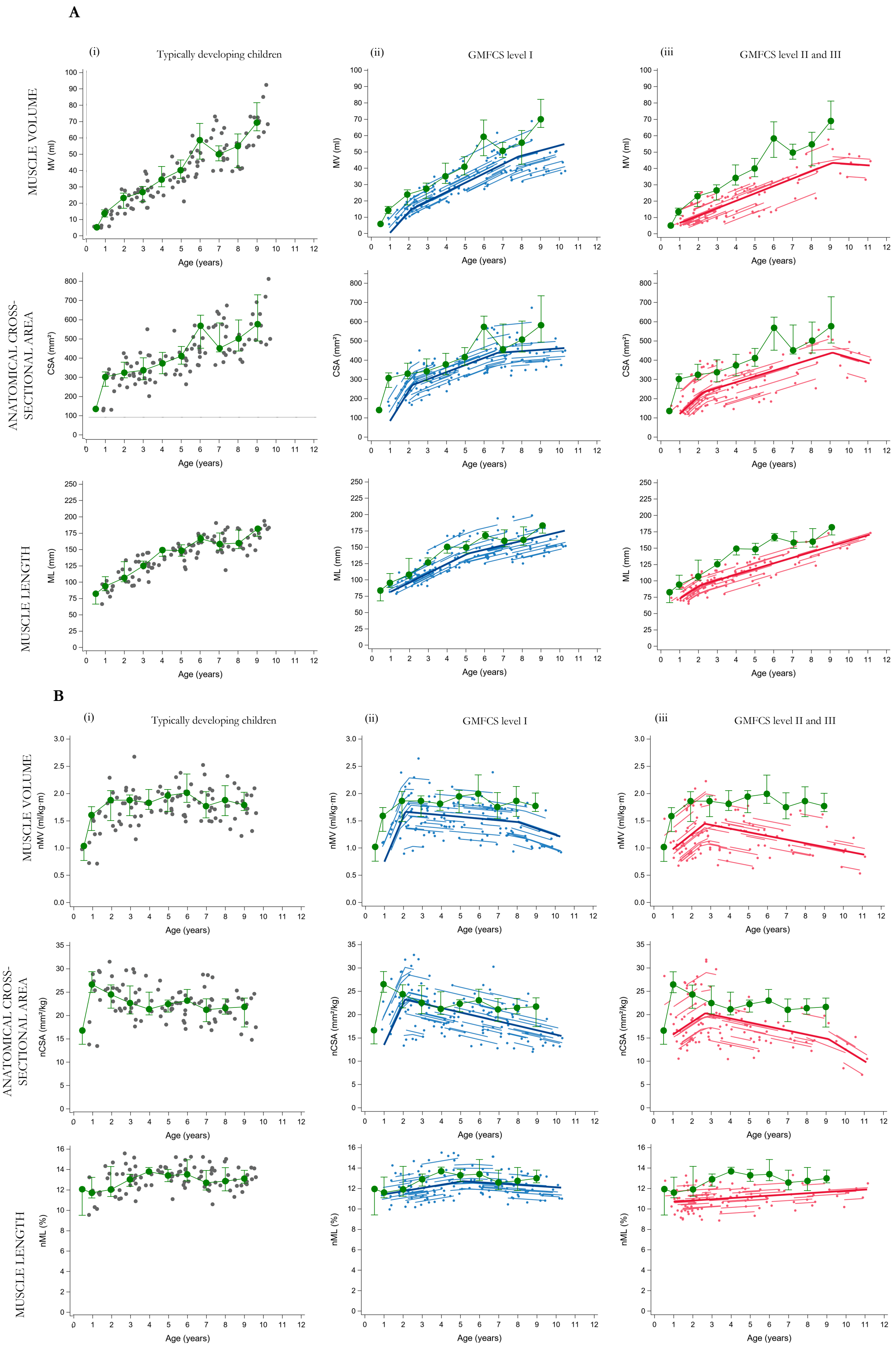
A
(i)
Typically developing children
(ii)
GMFCS level I
(iii)
GMFCS level II and III
MUSCLE VOLUME
MV (ml)
ANATOMICAL CROSS-SECTIONAL AREA
CSA (mm²)
MUSCLE LENGTH
ML (mm)
Age (years)
B
(i)
Typically developing children
(ii)
GMFCS level I
(iii)
GMFCS level II and III
MUSCLE VOLUME
nMV (ml/kg·m)
ANATOMICAL CROSS-SECTIONAL AREA
nCSA (mm²/kg)
MUSCLE LENGTH
nML (%)
Age (years)

Table S1

Muscle morphology of retrospective cross-sectional dataset with TD children.

Outcome	Number of children	**Median (IQ1-IQ3)**
Age (y)	102	5.2 (3.0-7.3)
Body mass (kg)	102	18.7 (14.5-23.0)
Body length (cm)	102	110.3 (93.4-123.3)
MV (mL)	102	38.7 (24.7-123.3)
nMV (mL/kg·m)	102	1.8 (1.6-2.0)
CSA (mm^2)	100	409.3 (327.1-501.2)
nCSA (mm^2/kg)	100	22.6 (20.6-25.2)
ML (mm)	102	145.4 (121.9-161.2)
nML (%)	102	13.0 (12.0-13.8)

Anthropometric and muscle data are presented as median (interquartile 1- interquartile 3). *TD, typical developing children;* IQ, interquartile; *y, years; kg, kilogram; cm, centimeter; MV, muscle volume; mL, milliliter; n, normalized; CSA, anatomical cross-sectional area; ML, muscle belly length; kg, kilogram; m, meters.*

Table S2 Standard clinical care of all SCP participants.

	**GMFCS level I group**	**GMFCS level II-III group**
Participants (n)	47	40
Use of orthoses during the day	Frequently used, *n* = 25	Frequently used, *n* = 22
	Not frequently used, *n* =12	Not frequently used, *n* = 11
	Insoles, *n*=2	
	Not used, *n* = 8	Not used, *n* = 7
Use of orthoses during the night	Frequently used, *n* = 5	Frequently used, *n* = 5
	Not frequently used, *n* = 3	Not frequently used, *n* = 1
	Not used, *n* = 39	Not used, *n* = 34
Physiotherapy, min per week	75 (15 to 300)	120 (15 to 300)
Oral medication	Tone reduction, *n* = 3	Tone reduction, *n* = 9
	Anti-epileptics, *n* = 3	Anti-epileptics, *n* = 6
	Others, *n* = 6	Others, *n* = 14
	Vitamin D-supplement, *n*= 7	Vitamin D-supplement, *n*= 11
	Not used, *n* = 29	Not used, *n* = 12
BoNT-A treatment in the MG muscle during follow-up (n)	13	11
Serial casting during follow-up (n)	6	1

Frequently used indicated ≥ 50% of the day or night whereas not frequently used indicated <50% of the day or night. The amount of physiotherapy is shown as median (minimum to maximum values). *GMFCS, Gross Motor Function Classification System; n, number of children; BoNT-A, botulinum neurotoxin type A; MG, medial gastrocnemius.*

Table S3

Fixed and random effects of the piecewise regressions for muscle morphology (GMFCS I n=47 and GMFCS II-III n=40).

		**Intercept**	Breakpoints (c)		Variance random intercept	Variance residual
Outcome	Participants	α_0 (CI) *p-value*	c_1 (CI) *p-value*	c_2(CI) *p-value*	σ^2 (=a_{1i}) (CI) *p-value*	σ^2(=$\varepsilon_{(1)ij}$) (CI) *p-value*
MV	GMFCS I	13.2 (10.6-15.7) ***<0.0001***	2.1 (1.8-2.5) ***<0.0001***	7.8 (6.6-9.0) ***<0.0001***	6.7 (5.4-7.9) ***<0.0001***	2.8 (2.5-3-2) ***<0.0001***
	GMFCS II-III	11.1 (9.7-12.5) ***<0.0001***	9.1 (9.1-9.1) ***<0.0001***			
nMV	GMFCS I	1.57 (1.39-1.75) ***<0.0001***	2.1 (2.0-2.6) ***<0.0001***	8.0 (8.0-8.0) ***<0.0001***	0.3 (0.3-0.3) ***<0.0001***	0.1 (0.1-0.2) ***<0.0001***
	GMFCS II-III	1.25 (1.12-1.39) ***<0.0001***	2.7 (2.3-3.1) ***<0.0001***			
CSA	GMFCS I	241.1 (209.2-273.0) ***<0.0001***	2.2 (2.0-2.4) ***<0.0001***	6.7 (5.6-7.8) ***<0.0001***	62.0 (50.6-73.3) ***<0.0001***	39.3 (32.6-46.0) ***<0.0001***
	GMFCS II-III	209.5 (172.7-246.2) **<0.0001**	2.7 (1.5-3.0) ***<0.0001***	9.1 (8.0-10.3) **<0.0001**		
nCSA	GMFCS I	22.4 (20.3-24.4) ***<0.0001***	2.1 (2.1-2.1) ***<0.0001***	9.1 (7.7-10.6) ***<0.0001***	3.7 (3.0-4.4) ***<0.0001***	2.4 (2.0-2.9) ***<0.0001***
	GMFCS II-III	18.4 (16.4-20.4) ***<0.0001***	2.7 (2.0-3.5) ***<0.0001***			
ML	GMFCS I	95.9 (92.2-99.6) ***<0.0001***	5.1 (4.5-5.8) ***<0.0001***		12.9 (10.1-15.7) ***<0.0001***	6.3 (5.1-7.5) ***<0.0001***
	GMFCS II-III	91.3 (86.2-96.4) ***<0.0001***	2.1 (1.6-2.6) ***<0.0001***			
nML	GMFCS I	11.7 (11.3-12.2) ***<0.0001***	5.1 (4.0-6.2) ***<0.0001***		0.8 (0.7-1.1) ***<0.0001***	0.7 (0.6-0.8) ***<0.0001***
	GMFCS II-III	10.8 (10.5-11.2) ***<0.0001***				

Results of the differences between slopes and breakpoints (GMFCS, I n=47, and GMFCS II-III n=40).

Outcome	**GMFCS I**	*Δ*	*p-value*	**GMFCS II-III**	*Δ*	*p-value*
MV	$\beta_{1,I}$ vs. $\beta_{2,I}$	7.08	**0.0021**	$\beta_{1,II\text{-}III}$ vs. $\beta_{2,II\text{-}III}$	5.32	**0.0255**
	$\beta_{2,I}$ vs. $\beta_{3,I}$	2.57	**0.0278**			
	$\beta_{1,I}$ vs. $\beta_{3,I}$	9.65	**0.0001**			
nMV	$\beta_{1,I}$ vs. $\beta_{2,I}$	0.86	**<0.0001**	$\beta_{1,II\text{-}III}$ vs. $\beta_{2,II\text{-}III}$	0.35	**<0.0001**
	$\beta_{2,I}$ vs. $\beta_{3,I}$	0.09	**0.0222**			
	$\beta_{1,I}$ vs. $\beta_{3,I}$	0.95	**<0.0001**			
CSA	$\beta_{1,I}$ vs. $\beta_{2,I}$	121.4	**<0.0001**	$\beta_{1,II\text{-}III}$ vs. $\beta_{2,II\text{-}III}$	58.8	**0.0050**
	$\beta_{2,I}$ vs. $\beta_{3,I}$	30.0	**0.0095**	$\beta_{2,II\text{-}III}$ vs. $\beta_{3,II\text{-}III}$	58.1	**0.0067**
	$\beta_{1,I}$ vs. $\beta_{3,I}$	151.4	**<0.0001**	$\beta_{1,II\text{-}III}$ vs. $\beta_{3,II\text{-}III}$	116.9	**0.0112**
nCSA	$\beta_{1,I}$ vs. $\beta_{2,I}$	9.83	**<0.0001**	$\beta_{1,II\text{-}III}$ vs. $\beta_{2,II\text{-}III}$	3.57	**0.0106**
				$\beta_{2,II\text{-}III}$ vs. $\beta_{3,II\text{-}III}$	1.65	**0.0424**
				$\beta_{1,II\text{-}III}$ vs. $\beta_{3,II\text{-}III}$	5.21	**0.0005**
ML	$\beta_{1,I}$ vs. $\beta_{2,I}$	7.43	**<0.0001**	$\beta_{1,II\text{-}III}$ vs. $\beta_{2,II\text{-}III}$	10.3	**0.0089**
nML	$\beta_{1,I}$ vs. $\beta_{2,I}$	0.43	**0.0117**	NA		

P-values in bold indicate significance level at $p<0.05$. The following symbols represent: α = response at the age of 2 years, CI= 95% confidence interval, c = age (years) of the breakpoint, $\sigma^2(a_{1i})$ = variance of random intercept and $\sigma^2(\varepsilon_{(1)ij})$= variance of residual, β = change in outcome per year and Δ = difference score. *GMFCS, gross motor function classification system; n, number; MV, muscle volume; n, normalized; CSA, anatomical cross-sectional area; ML, muscle belly length; NA, not applicable.*
